# Association between menstrual pad usage, self-reported symptoms, and menstrual blood loss measured by the alkaline hematin method

**DOI:** 10.1016/j.ejogrb.2025.114536

**Published:** 2025-06-17

**Authors:** Jacqueline Fahey, Ana Cachau-Hansgardh, Ram K. Parvataneni, L.Elaine Waetjen, Jennifer C. Fung, Vanessa L. Jacoby

**Affiliations:** a David Geffen School of Medicine at UCLA, Department of Obstetrics and Gynecology, 10833 Le Conte Ave, Los Angeles, CA 90095, USA; b TheraNova LLC, 255 Kansas St, Suite 300, San Francisco, CA 94103, USA; c UC Davis School of Medicine, Department of Obstetrics and Gynecology, 4610 X St, Sacramento, CA 95817, USA; d UC San Francisco School of Medicine, Department of Obstetrics, Gynecology, and Reproductive Sciences, 533 Parnassus Ave, San Francisco, CA 94143, USA

**Keywords:** Heavy menstrual bleeding, Pad usage, Self-reported symptoms

## Abstract

**Background:**

Accurate assessment of heavy menstrual bleeding is necessary to determine effectiveness of therapeutic interventions. There are limited quantitative methods for evaluating heavy menstrual bleeding, as the validated alkaline hematin method has practical limitations, so alternative methods are needed.

**Objective:**

To evaluate the correlation between self-reported heavy menstrual bleeding, pad usage, and menstrual blood loss measured using the alkaline hematin method.

**Study Design:**

In this multicenter, prospective cohort study, 79 participants aged 18–50 years, who self-reported heavy menstrual bleeding, were recruited from February to November 2023. Participants provided demographic and medical history details and used study-provided pads during one menstrual cycle. Study outcomes were number of pads used per cycle and quantified menstrual blood loss measured using the alkaline hematin method. Data were analyzed using descriptive statistics.

**Results:**

The median menstrual blood loss per cycle was 66.1 mL (IQR 28.7–122.5 mL), with only 25.3 % (20/79) of participants exceeding the heavy menstrual bleeding threshold of 120 mL. Participants with heavy menstrual bleeding used a median of 21.5 pads per cycle (IQR 15.8–25.3 pads) while those with normal menstrual blood loss only used 11 pads per cycle (IQR 8–15 pads).

**Conclusion:**

This study found a discrepancy between self-reported and objectively measured heavy menstrual bleeding. Heavy menstrual bleeding is associated with greater pad usage per cycle, however there is overlap in pad usage between participants with normal and heavy menstrual bleeding. Accurate quantification of menstrual blood is crucial for clinical management and research, as self-reported measures may not reliably identify heavy menstrual bleeding.

## Introduction

Abnormal uterine bleeding (AUB) is a distressing medical condition that affects up to 30 % of women and transgender men of reproductive age, and is a heightened concern in perimenopause [1,2]. In addition to the negative impact on patient quality of life, AUB leads to significant economic productivity loss and an estimated annual direct economic cost as large as $1 billion [2,3]. It represents one of the most common gynecological problems and accounts for approximately one-third of gynecology office visits [1].

AUB is an abnormality of at least one component of menstrual bleeding, including cycle frequency, duration or volume [4]. Heavy menstrual bleeding (HMB) is part of the evaluation for AUB, and historically was quantified as menstrual blood loss (MBL) more than 80 mL per cycle [4]. Recent literature has demonstrated the risk of developing anemia is not significantly increased until patients exceed 120 ml of menstrual blood loss in one cycle, suggesting this as a more clinically meaningful threshold for HMB [5]. The proposed clinical definition of HMB is “excessive menstrual blood loss that interferes with a patient’s physical, social, emotional, and/or material quality of life [6,7].”.

While patient reports are essential for understanding their experiences and care goals, accurate assessment of HMB remains crucial to determine effectiveness of treatment interventions and to guide clinical decisions, yet there are limited quantitative methods for evaluating it. The current benchmark for measurement of MBL in research settings is the validated alkaline hematin (AH) method, however the AH method has practical limitations and restricted accessibility [8,9]. As a result, alternative approaches to evaluate MBL have been developed, including self-reported duration of bleeding, pad usage, and pictorial methods [8]. However, the accuracy of these methods is uncertain.

Pad usage has the potential to serve as a fast and simple approach to diagnose HMB. In this study, MBL data was collected from participants who self-reported HMB to determine whether pad usage correlates with measured HMB based on the validated AH method. Quality of life data was also collected to help understand whether a more nuanced and patient-centered approach that includes the impact of MBL on quality of life can be used to assess for HMB. Finally, post-menstrual cycle hemoglobin data was collected to evaluate whether chronic anemia at the expected point of hemoglobin nadir is associated with measured HMB.

## Materials and methods

This was a multicenter, prospective cohort study conducted at three large academic institutions. IRB approval was obtained from each site. From February to November 2023, participants were recruited locally at the three study sites through advertising. Participants were required to be 18–50 years old, speak English, have at least 2 menses in the past 3 months, and self-report HMB. An ability to capture images with a provided smartphone for the duration of one menstrual cycle was also required for study eligibility. Exclusion criteria included current or prior pregnancy within the past 6 months, or intention to become pregnant or undergo a gynecologic procedure during the study period. Participants were also excluded if they had prolonged menstrual bleeding lasting 10 days or longer in the past 6 months, active cancer, or a known diagnosis of human immunodeficiency virus, hepatitis B or hepatitis C. For eligible participants, informed consent was obtained. All study procedures were performed in compliance with institutional guidelines and were approved by the site-specific institutional committees.

Participants attended 2 study visits: an initial training and enrollment visit, and a follow-up visit after their menstrual cycle. During the initial visit, participants signed the informed consent form, underwent a urine pregnancy test, and provided demographic characteristics and medical history details. They also completed the Menstrual Bleeding Questionnaire (MBQ), which is a validated one-month retrospective self-reported outcome measure for MBL. With a total possible score of 0 to 75, the MBQ includes 20 items related to menstrual bleeding heaviness, cycle frequency, pain, and quality of life [10]. Any participant who did not have menstrual bleeding during the month prior to their enrollment visit did not complete the MBQ, and therefore did not receive a score. Participants then underwent in person and video training for proper data collection of menstrual pad usage. Finally, they received the study materials, including a supply of 2 commercially available menstrual pad products (Always^®^ Long and Overnight Pads), supply of photo mats, a study smartphone, and an airtight specimen collection kit for used menstrual pad storage and transport. During their next menstrual cycle, participants captured images of each used menstrual pad and entered this data using a smartphone mobile application designed for the study. Participants were instructed to only use the provided menstrual pads. Once their menstrual cycle was complete, they returned all study materials, including used menstrual pads, to the study site. Participants were instructed to self-report any missed entries when a pad was inadvertently discarded without capturing an image or returning the used pad to the study site.

At the follow-up visit, hemoglobin concentration was measured from venous blood samples and the used menstrual pads were processed using the AH method to quantify blood loss, with samples sent to an independent lab, Fung Lab UCSF. The AH method chemically measures blood volume of a menstrual pad and is considered the current gold standard for determining MBL [11,12]. Using the AH method, blood loss was estimated as follows: absorbance values were normalized by subtracting the absorbance of an unused menstrual pad. Normalized absorbance was converted to hemoglobin mass using menstrual pad-specific conversion factors. These conversion factors were determined through benchtop recovery experiments by applying known volumes of blood to each pad type and characterizing the relationship between absorbance and hemoglobin mass. Finally, estimated blood loss volume was calculated from hemoglobin mass and participant-specific hemoglobin concentration.

The outcomes were the total number of used menstrual pads as documented by the participant using digital photos, measurement of MBL in milliliters (mL) using the AH method during one menstrual cycle, pre-menstrual cycle MBQ score, and post-menstrual cycle hemoglobin (Hgb) concentration in grams per deciliter (g/dL). The data used to examine these outcomes was a secondary analysis of data that had been collected for a study aimed at validating a new electronic method for estimating MBL. As such, the sample size was determined by the existing dataset from the original study. Given the relatively small cohort, descriptive statistics were primarily used to summarize the data. Median and interquartile range (IQR) were calculated for discrete and continuous variables, with the median included in the IQR. All analyses were conducted using standard spreadsheet software.

## Results

**Fig. 1.**
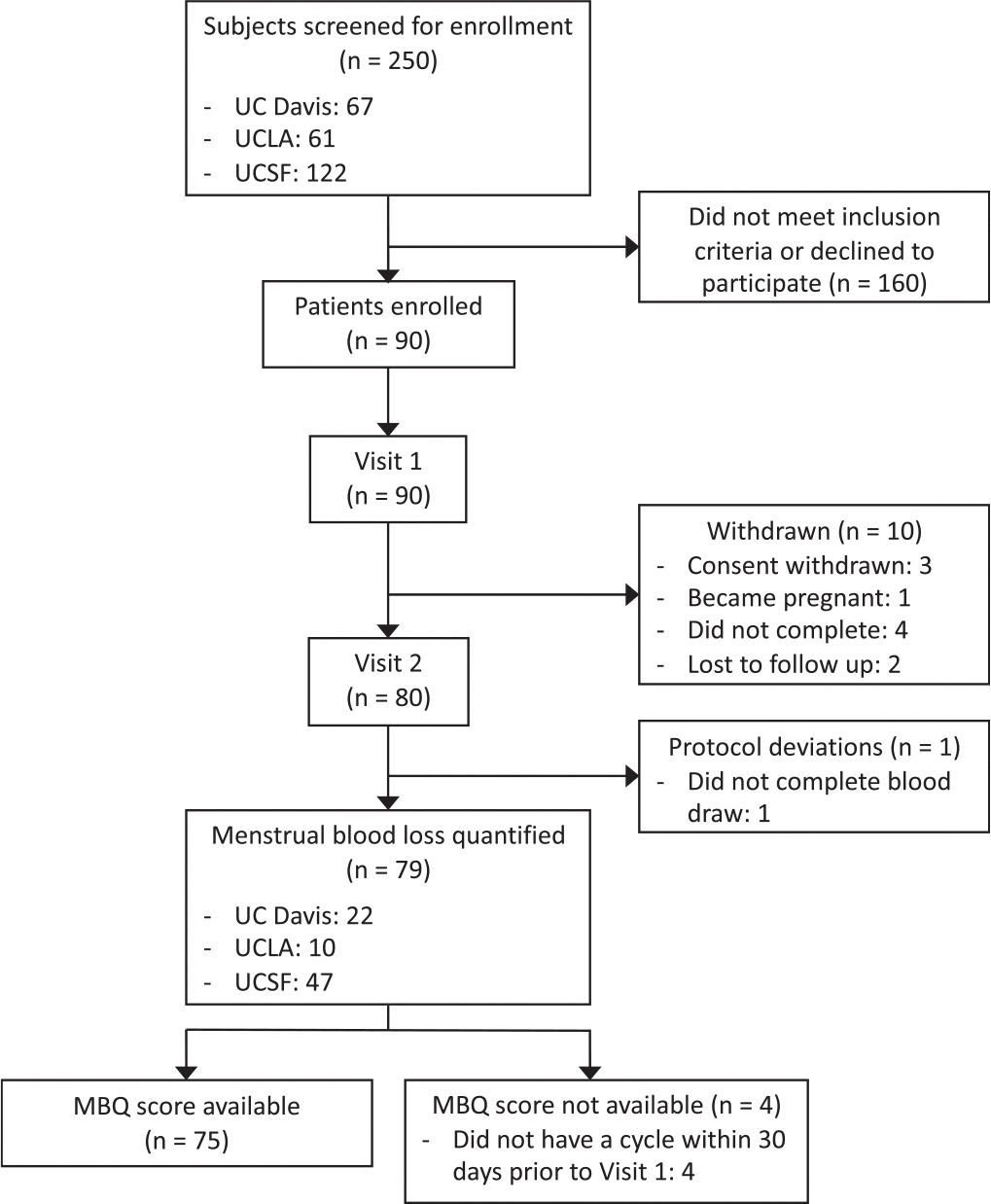
Recruitment flowchart for a multicenter, observational cohort study quantifying menstrual blood loss (MBL).

A total of 250 participants were screened for eligibility. One hundred and sixty participants did not meet inclusion criteria or declined to participate. Ninety participants were enrolled in the study, but 10 participants were withdrawn and 1 participant had a protocol deviation due to a missed blood test, resulting in 79 participants’ data available for analysis who completed the initial visit, menstrual pad collection, and follow-up visit (Fig. 1). Specific reasons that 11 enrolled participants did not complete the study included withdrawn consent, pregnancy, failure to complete menstrual pad collection, lost to follow-up, or incomplete blood draw. A total of 1090 valid pad samples with complete AH were included in the data analysis. There were 46 unaccounted pad samples that were not included in data analysis due to image taken but no sample returned (n = 7), invalid pad returned (n = 2), or self-reported missed entry (n = 37).

Table 1 summarizes participant and menstrual cycle characteristics. Their ages ranged from 19 to 50 years old (median 39 years old). BMI was evenly distributed between the normal (27.8 %), overweight (38.0 %), and obese (34.2 %) categories. Most participants were either White (38.0 %), Asian (20.3 %), or Black race (13.9 %). About one quarter of participants (27.8 %) were of Hispanic or Latina ethnicity. Participants used a median of 12 pads per cycle (IQR 8–19). Using the AH method, median MBL was 66.1 mL per cycle (IQR 28.3–119.3 mL). Only 25.3 % of participants met blood loss criteria for HMB diagnosis, defined as at least 120 mL MBL per cycle. The range of blood volume per pad was 0.0 to 38.1 mL, with a median of 8.7 mL per pad in participants with HMB and 3.6 mL per pad in those with normal MBL.

**Fig. 2.**
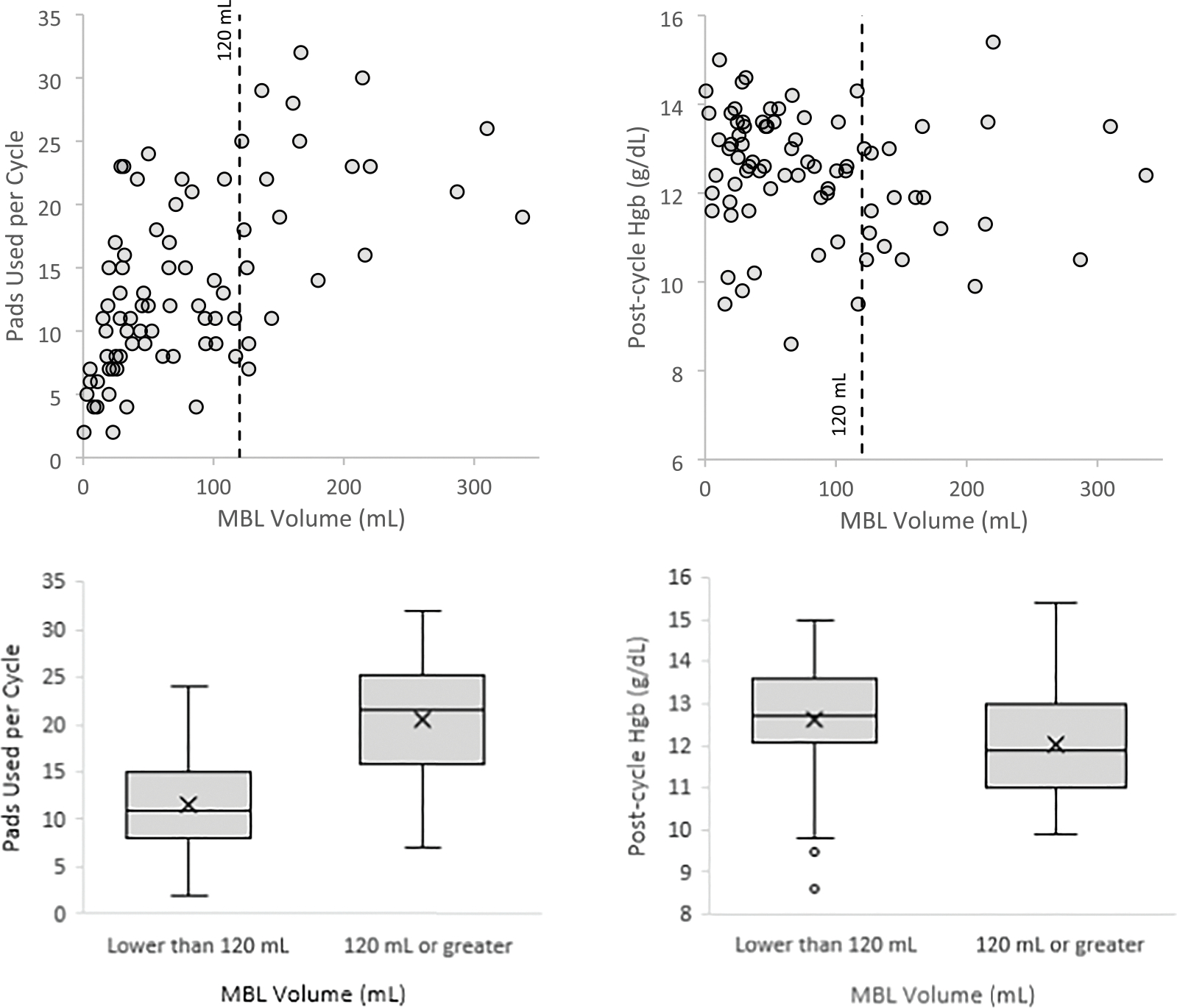
Pads used and post-cycle hemoglobin (Hgb) versus menstrual blood loss (MBL) per cycle.

**Fig. 3.**
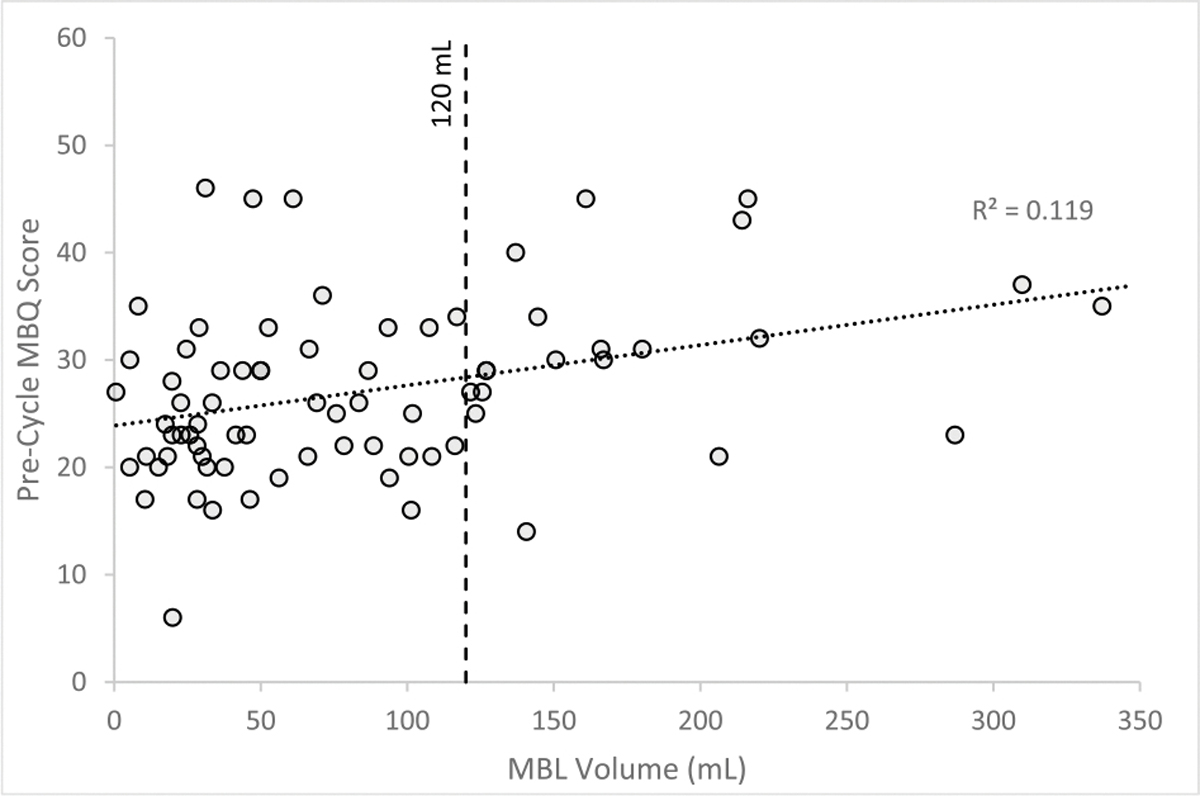
Pre-cycle menstrual bleeding questionnaire (MBQ) score versus menstrual blood loss (MBL) per cycle.

Fig. 2 shows pad usage and post-cycle Hgb by menstrual blood loss. Participants with HMB used a median of 21.5 pads per cycle (IQR 15.8–25.3 pads) while those with normal MBL only used 11 pads per cycle (IQR 8–15 pads). All participants who had normal MBL used less than 25 pads per cycle. Median post-cycle Hgb was 11.9 g/dL for participants with HMB and 12.7 g/dL for those with normal MBL, indicating the median post-cycle Hgb for those with HMB met WHO criteria for anemia (Hgb < 12 g/dL), although the variation in post-cycle Hgb was wider for the HMB group [13]. There was a weak correlation (R^2^ = 0.119) between pre-cycle MBQ score and MBL per cycle (Fig. 3). Among the 75 participants who experienced bleeding in the month prior to their initial visit, the median pre-cycle MBQ score was 26 (IQR 21–31) (Table 2).

### Comment

#### Principal findings

A discrepancy exists between self-reported and quantified HMB, as all participants in this study endorsed HMB, while only one quarter met diagnostic criteria, measured as at least 120 mL MBL per cycle using the AH method. HMB was associated with greater median pad usage per cycle and MBL volume per pad. However, there was wide variation and overlap in pad usage between participants with normal MBL and HMB, which supports existing literature that indicates subjective measures of MBL are suboptimal in comparison to the AH method [8].

#### Results in the context of what is known

Although the AH method is the gold standard for quantifying MBL in research settings, it is difficult to use in clinical practice because it involves specific calibration for each type of sanitary product, requires patients to collect and send sanitary products for evaluation, and fails to account for the range of adverse effects menstrual bleeding may have on a patient’s quality of life [8,9]. In an effort to address these limitations, other approaches to assessing HMB have been developed such as self-reported HMB, menstrual pad counts, and pictorial methods like the pictorial blood loss assessment chart (PBAC) [8]. While such subjective methods have the advantage of being quick and simple assessments, they do not precisely measure MBL and have not reliably been shown to diagnose HMB [8]. Using an objective method to assess for anemia has the potential to be a rapid and clinically meaningful approach to evaluating HMB, however post-cycle Hgb for participants in this study varied widely.

Several menstrual questionnaires have been developed to evaluate HMB and its impact on quality of life, although these questionnaires have generally not been as extensively validated to diagnose HMB [8,9]. The validated MBQ has particular advantages over other menstrual questionnaires in that it accounts not only for heaviness and duration of bleeding, but also other aspects of menstruation that may negatively impact quality of life, including limitations in activities, predictability, and social embarrassment [8–10]. It has been shown that the MBQ, which only asks participants to report menstrual symptoms over the prior 1 month, is not affected by recall bias [9,10]. In the validation study, the MBQ was able to discriminate between women with and without self-reported HMB, as there was a significant difference in mean MBQ score between the two groups (mean 30.8 with self-reported HMB vs 10.6 without self-reported HMB, P < 0.0001) [10]. Subsequent studies have suggested MBQ scores ranging from 21.5 to 24 as a diagnostic cut off for HMB [14,15]. Despite these strengths, in this study there was not a strong correlation between MBQ scores and measured MBL volume on the subsequent cycle.

The most widely used alternative to the AH method for discriminating between normal MBL and HMB is PBAC. Using AH as the gold standard, PBAC has been shown to have a superior positive predictive value for HMB when compared to both self-reported HMB and laboratory-confirmed anemia [16]. More recent systematic reviews of PBAC, however, demonstrated mixed results with regards to its sensitivity and specificity for detecting HMB [17,18]. Moreover, PBAC is subject-scored, relying on the participants’ visual estimate and interpretation of the blood on sanitary products, so this semi-objective assessment does not precisely quantify MBL.

#### Clinical implications

Accurate assessment of MBL is clinically important as it informs decisions about medical and surgical interventions [8]. There are practical limitations of the AH method in clinical settings, so subjective methods to assess HMB are often utilized. Self-reported symptoms alone do not reliably indicate which patients have HMB. Heavy menstrual bleeding is associated with greater pad usage per cycle, however there is overlap between participants with normal MBL and HMB, so this has limited utility as an indicator of HMB. Although there was not a strong correlation between MBQ score and objectively measured HMB in this study among women who all self-reported HMB, it could potentially still be used in this population to assess therapeutic response by comparing MBQ scores before and after treatment.

#### Research implications

Difference in MBL is regularly used to evaluate response to treatment interventions in clinical trials [8]. This study underscores the imprecise nature of self-reported HMB, menstrual pad usage, or MBQ scores when evaluating for HMB. Although PBAC may be an alternative method to assess effectiveness of therapeutic interventions for HMB, it does not quantify MBL. Ultimately a more practical and accessible objective measure of MBL should be developed and validated for use in research settings, as it could more readily be used than the AH method to supplement the established core outcome set, which outlines 10 other variables for use in clinical trials of interventions for HMB [19].

#### Strengths and limitations

Strengths of this study include a systematic training of participants to ensure consistency of menstrual pad data collection, use of standardized sanitary products, collection of pad count data prospectively using participant captured images of each menstrual pad, and independent lab quantitative analysis of MBL. Potential limitations include inability to account for factors that could not be captured in one menstrual cycle, such as health status changes and menstrual cycle variability. Additionally, the pre-cycle MBQ scores reflect one cycle prior to the cycle when objective MBL data was collected. Another limitation is the potential for underestimation of MBL due to unaccounted for blood loss external to the menstrual pad, such as MBL while bathing or toileting. The total number of participants was small and there may be a selection bias given the relatively large number of screened patients who were not enrolled, some of whom declined to participate after learning of the study requirements.

## Conclusion

Assessment of MBL with a quick and simple subjective measures such as self-reported HMB or pad usage per cycle may be of limited utility for clinical decisions regarding medical and surgical management of HMB. While subjective reports are important to understanding perceived impact of HMB, to precisely evaluate treatment response when interventions are used for HMB in both clinical and research settings, development of a more accessible objective measure of MBL than the AH method is a priority for the prevalent and distressing condition of HMB.

## Figures and Tables

**Table 1:** Participant and Menstrual Cycle Characteristics.

Characteristic	Total (n = 79)

Age (y)	39.0 (32.0–42.5)
BMI (kg/m^2^)*	28.3 (24.3–32.2)
Lower than 18.5	0 (0.0)
18.5–24.9	22 (27.8)
25–29.9	30 (38.0)
30 or higher	27 (34.2)
Race	
American Indian or Alaska Native	2 (2.5)
Asian	16 (20.3)
Black	11 (13.9)
Native Hawaiian/Pacific Islander	1 (1.3)
Two or more	8 (10.1)
White	30 (38.0)
Unknown/Decline to State	11 (13.9)
Ethnicity	
Hispanic or Latino	22 (27.8)
Not Hispanic or Latino	52 (65.8)
Unknown/Decline to State	5 (6.3)
Pads used per cycle	12 (8–19)
MBL volume (mL)^†^	61.1 (28.3–119.3)
MBL volume per pad (mL)	3.5 (0.9–8.3)
Post-cycle Hgb (g/dL)^‡^	12.6 (11.7–13.5)
Participants with MBL ≥ 120 mL	20 (25.3)
Participants with MBL < 120 mL	59 (74.7)

Data are median (interquartile range) or n (%).

* BMI, body mass index. BMI values are based on self-reported height and weight.

† MBL, menstrual blood loss.

‡ Hgb, hemoglobin.

**Table 2:** Scores of the Menstrual Bleeding Questionnaire (MBQ) Across Different Categories and Menstrual Blood Loss (MBL) Volumes.

Question	Total (n = 75)	MBL < 120 mL (n = 55)	MBL ≥ 120 mL (n = 20)

Heaviness and duration of bleeding			
1	3 (3–4)	3 (3–4)	3 (3–4)
2	2 (1–2)	2 (1–2)	2 (1–2.3)
3	1 (0–2)	1 (0–2)	2 (1–2.3)
4	1 (1–1)	1 (1–1)	1 (1–1)
5	1 (1–1)	1 (1–1)	1 (0–1)
6	1 (1–2)	1 (1–2)	1 (1–2)
7	0 (0–1)	0 (0–1)	0 (0–1)
8	2 (1–2.5)	2 (1–3)	2 (1–2)
9	0 (0–0)	0 (0–0)	0 (0–0)
**Subtotal (1 to 9)**	**12 (10–15)**	**12 (10–15)**	**11 (10–15)**
Limitations in activities			
10*	1 (0–1.5)	1 (0–2)	1 (1–1)
11*	1 (0–1.25)	1 (0–2)	1 (1–1)
12	1 (0–1)	1 (0–1)	1 (0–1)
13	1 (0–2)	1 (1–2)	1.5 (1–2)
14	1 (0–1)	1 (0–1)	1 (0–1)
15	1 (0–1)	1 (1–1)	0.5 (0–1)
**Subtotal (10 to 15)**	**4 (2.5–5)**	**4 (3–5)**	**3.5 (2–5)**
Social embarrassment			
16	0 (0–1)	0 (0–1)	0 (0–1)
17	1 (1–2)	1 (1–2)	1 (1–2)
18	7 (4.5–8)	6 (4.5–8)	7.5 (4.8–9)
**Subtotal (16 to 18)**	**9 (6–10)**	**9 (6–10)**	**9 (6.8–11)**
Predictability			
19	1 (0–1)	1 (0–1)	1 (0–1)
20	1 (1–1)	1 (1–1)	1 (1–1.25)
**Subtotal (19 to 20)**	**2 (1–2)**	**2 (1–2)**	**2 (1–2)**
**Total Pre-Cycle MBQ Score**	**26 (21–31)**	**26 (21–32.5)**	**26 (22.8–29.3)**

Data are median (interquartile range).

* For Question 10 and 11, scores were imputed for 12 respondents who were unable to answer because they were not working outside the home. Among these, 3 had MBL ≥ 120 mL, and 9 had MBL < 120 mL.
